# Influence of the Catalyst Particle Size on the Aqueous Phase Reforming of *n*-Butanol Over Rh/ZrO_2_

**DOI:** 10.3389/fchem.2020.00017

**Published:** 2020-01-28

**Authors:** Heikki Harju, Giuseppe Pipitone, Leon Lefferts

**Affiliations:** ^1^Department of Chemical and Metallurgical Engineering, Aalto University, Espoo, Finland; ^2^Catalytic Processes and Materials, Department of Science and Technology, MESA+ Institute for Nanotechnology, University of Twente, Enschede, Netherlands; ^3^Department of Applied Science and Technology, Politecnico di Torino, Turin, Italy

**Keywords:** aqueous phase reforming, hydrogen, mass transfer, reaction pathway, rhodium

## Abstract

Butanol is a by-product obtained from biomass that can be valorized through aqueous phase reforming. Rh/ZrO_2_ catalysts were prepared and characterized, varying the size of the support particles. The results showed a relatively mild effect of internal mass transport on butanol conversion. However, the influence of internal transport limitations on the product distribution was much stronger, promoting consecutive reactions, i.e., dehydrogenation, hydrogenolysis, and reforming of propane and ethane. Hydrogen consuming reactions, i.e., hydrogenolysis, were more strongly enhanced than hydrogen producing reactions due to internal concentration gradients. Large support particles deactivated faster, attributed to high concentrations of butyraldehyde inside the catalyst particles, enhancing deposit formation via aldol condensation reactions. Consequently, also the local butyric acid concentration was high, decreasing the local pH, enhancing Rh leaching. The influence of internal transfer limitation on product distribution and stability is discussed based on a reaction scheme with three main stages, i.e., (1) formation of liquid intermediates via dehydrogenation, (2) formation of gas via decarbonylation/decarboxylation reactions, and (3) hydrocarbon hydrogenolysis/reforming/dehydrogenation.

## Introduction

The environmental issues and the depletion of conventional sources of energy demand development of alternative and sustainable technologies. Among the several possibilities, biomass is seen as a strategic feedstock for the production of renewable energy and materials. One of the possible products of biomass exploitation is hydrogen, through thermochemical or biological routes (Balat and Kirtay, 2010).

In the last years, a considerable effort has been put on the production of hydrogen from oxygenated hydrocarbons, e.g., via aqueous phase reforming (APR) (Cortright et al., 2002). The Dumesic research group demonstrated that hydrogen can be produced from alcohols in water in the condensed phase, with a noteworthy energetic advantage compared to the conventional steam reforming as evaporation of water is circumvented (Davda et al., 2005).

APR is a promising strategy for valorization of aqueous side-streams. Among the possible reactants for this process, oxygenates with 1 to 1 O to C ratios are preferred for H_2_ production, via reforming and subsequent water gas shift reaction (Cortright et al., 2002; Shabaker et al., 2003a; Davda et al., 2005). Indeed, methanol is the most investigated mono-alcohol, thanks to its optimal carbon/oxygen ratio.

However, little attention has been paid so far to APR of butanol, despite its strategic importance as it can be produced from biomass via fermentation of sugar cane (Kumar et al., 2017). Also, aqueous waste streams of e.g., flash pyrolysis contain butanol, making it an interesting model compound for study. As a matter of fact, butanol has been studied for hydrogen production vis supercritical water reforming for its representativeness of oxygenates present in the bio-oil aqueous phase (Gutiérrez Ortiz et al., 2016; Gutiérrez Ortiz and Campanario, 2018).

Roy et al. investigated for the first time APR of butanol over Ni-based catalysts supported on ceria or alumina (Roy et al., 2011). Successively, the same research group used these catalysts also in harsher conditions, i.e., for steam reforming of butanol, enlarging the range of operating conditions as reaction temperatures, pressure, concentration, and flow rate of the feed (Roy et al., 2014).

In previous work we investigated the steam reforming of butanol over a Rh/ZrO_2_ catalyst (Harju et al., 2015, 2016). This catalytic system showed promising results in terms of hydrogen productivity and stability, since coke formation is slower compared to other supports. APR of higher alcohols results in significant formation of hydrocarbons on more conventional metal catalysts (Roy et al., 2011, 2014; Lobo et al., 2012) because of limited C-C cleavage. Rh however is known to be active for C-C cleavage (Sinfelt, 1973; Bond et al., 1996) and low temperature steam reforming (Kolb et al., 2004; Halabi et al., 2010). It has been recently showed that, among several metals (Pd, Ru, Re, Ir, and Cr) Rh was the best promoter in a bimetallic Pt-based catalyst for the APR of glycerol (Larimi and Khorasheh, 2019). Furthermore, ZrO_2_ is known as one of the few oxides capable of resisting the harsh hydrothermal conditions of APR (Elliott et al., 1993), unlike more commonly used supports (De Vlieger et al., 2012). Therefore, this study explores the use of Rh/ZrO_2_ for the valorization of butanol in APR conditions.

APR is a three phase (G-L-S) system and therefore issues related to mass transfer limitations may arise. Hydrogen mass transfer is of paramount importance in APR as reported by Neira D'Angelo et al. (2013, 2014a,b) reporting that microchannel reactors enhance mass transfer, increasing the hydrogen yield by suppressing sequential reactions consuming H_2_.

The goal of this work is to determine the effect of internal mass transfer on aqueous phase reforming of butanol over Rh/ZrO_2_ catalyst by varying the dimension of the catalyst particles using a reactor design that increase the sensitivity for internal mass transfer. For example, in contrast to previous works (Neira D'Angelo et al., 2013, 2014a), the reactor was not flushed with inert gas, so that the concentration of gas products outside the catalyst particles was relatively high, retarding diffusion out of gaseous products. The obtained results allow not only to discuss the effect of internal mass transport on product distribution and deactivation, but also to propose a reaction scheme.

## Materials and Methods

### Catalyst Synthesis

The catalysts were prepared by vacuum assisted dry impregnation method followed by calcination at 850°C, described in more detail in earlier work (Harju et al., 2015). To obtain the different catalyst particle sizes, the ZrO_2_ support (monoclinic ZrO_2_, MEL Chemicals) was crushed and sieved to the desired particle sizes prior to impregnation. The support particles sizes remained unchanged during catalyst preparation.

### Catalyst Characterization

The Rh content of both fresh and spent catalyst was determined by X-Ray Fluorescence performed with Malvern Panalytical Axios mAX 3 kW. The 7-point BET surface area of both fresh and spent catalyst was performed with Coulter Omnisorp 100 CX, using methods described elsewhere (Kaila et al., 2007, 2008). Pore volume of both fresh and spent catalyst were determined during the same N_2_ physisorption measurement as the BET surface area, using the NLDFT method, assuming spherical and cylindrical pores. Unfortunately, the equipment used for BET measurement did not allow experiments with small particles and only the samples with the largest particle size could be measured. The Rh dispersion on both fresh and spent catalyst was determined using pulsed H_2_ chemisorption in Ar (Chemisorb 2750, Micromeritics). The Rh particle size distribution on spent catalyst was determined by scanning tunneling electron microscopy (STEM) using Jeol 2200FS equipped with spherical aberration corrector. Elemental C, H, and O analysis of the deposits on spent catalyst was done using methods described in previous work (Harju et al., 2016). The elemental analysis was also done for fresh catalyst to determine the contribution of hydroxyl and carbonate groups of atmospheric origin.

### Reaction Experiments

**Figure 1:**
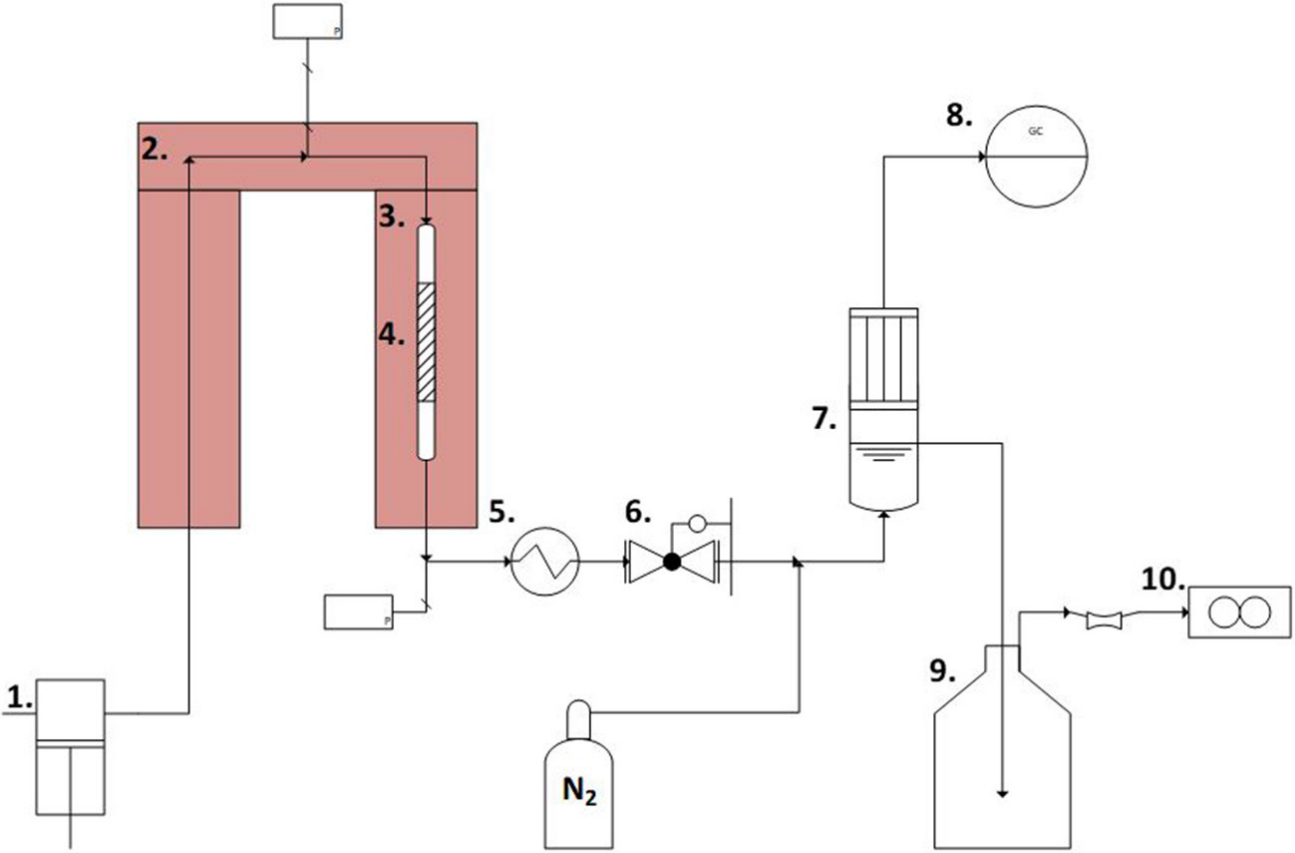
Schematic of the experimental set-up. 1. Piston pumps. 2. Pre-heater. 3. Heater. 4. Catalyst section. 5. Heat exchanger. 6. Back-pressure valve. 7. Gas-liquid separation vessel. 8. Varian Micro-GC. 9. Eluent collection vessel. 10. Gas mass-flow meter.

A schematic representation of the set-up used for APR experiments is reported in Figure 1. The reactant solution (5 wt% *n*-butanol in de-ionized water) was pressurized and fed by a setup of dual ISCO D-pumps and pre-heated up to the desired reaction temperature (220°C) before the catalytic bed. The catalytic bed was 7 mm in diameter. The catalyst loading in the reactor was varied (1.5, 1.0 and 0.1 g for the 250–420, 60–100, and 40–60 μm catalysts, respectively, resulting is acceptable bed height), keeping the LHSV constant at 150 h^−1^ for all the experiments by adjusting the volumetric flow rate. The set temperature was reached about 30 min after starting heating, and it was considered in the results as the zero time. Afterwards the effluent was cooled down to room temperature by a water-cooled tubular heat exchanger. The pressure was kept constant at ~35 bar by a manual back-pressure regulator (TESCOM, model 26-1764-24-090) following the heat exchanger. Prior to phase separation at ambient pressure in a separation vessel, nitrogen sweep gas was introduced to the stream to aid in gas product purging and to keep a steady minimum flow present to improve accuracy of both gas phase analysis and flow rate measurement. The gaseous products were periodically sampled from the top of the de-mister section of the phase separation vessel for analysis in a Varian micro-GC, while the gas flowrate was measured by a gas mass flow meter. The liquid flowrate was integrally measured by weighing the eluent collection vessel mounted on a scale.

The time on stream (TOS) was 150 min for the 40–60 and 60–100 μm particles and 210 min for the 250–420 μm particles. After cooling the system overnight, the catalyst was removed from the reactor and dried at 100°C overnight.

### Product Analysis and Calculation

The gas products were analyzed with a Varian micro-GC equipped with two columns with a TCD detector to determine the yields. H_2_, O_2_, N_2_, CH_4_, and CO were analyzed with a Molsieve 5A column (argon carrier, temperature column 100°C), while CO_2_, C_2_H_4_, C_2_H_6_, C_3_H_6_, and C_3_H_8_ were analyzed over a PoraPLOT U column (helium carrier, temperature column 85°C).

The liquid phase was sampled every 30 min and analyzed with a Shimadzu HPLC (Prominence) equipped with an Aminex HPX-87H column and a refractive index detector (RID). The flow rate of the mobile phase (5 mM H_2_SO_4_ aqueous solution) was fixed at 0.6 ml/min and the working temperature at 30°C. External calibration curves were used for the quantification. Liquid flow rates were measured according to the variation of the mass in the collecting vessel, assuming density of the liquid product equal to water at room temperature. Gas flow rates were measured with a Brooks gas flow meter; as it was calibrated for nitrogen, the actual value was obtained using conversion factors, knowing the gas composition. The gas flowrate out of the reactor was not constant, coming out in larger bursts, occasionally causing scatter in the micro-GC results and gas mass flow measurement. This problem occurs when gas bubbles form upstream of the backpressure regulator in case of high conversion and high gas-yields. These errors were mitigated by averaging over 30 min periods.

The liquid product yields (C1 based) were calculated according to Equation (1), where *Y*_*C*_1_, *i*_ is the C_1_ based yield of any carbon containing component *i, F*_*i*_ is the molar flow rate (mol min^−1^) of component *i, N*_*Ci*_ is the carbon number of component *i*. The gas product yields (C1 based) were measured according to Equation (2) as the difference between butanol conversion and liquid yields, based on the fact that the carbon mass balance closed within 10%. The gas selectivity was defined as the ratio between the gas product yield and the butanol conversion. Finally, the selectivity toward a specific component *i* was defined as the ratio between its molar flow and moles of reacted butanol (Equation 4).

(1) $$\begin{array}{l} Y_{C_{1},liquid}=\frac{\sum F_{i_{liquid}}N_{C_{i}}}{F_{butanol_{in}}N_{C_{butanol}}} \end{array}$$

(2) $$\begin{array}{l} Y_{C_{1},\;gas}=X_{butanol}-Y_{C_{1},\;liquid} \end{array}$$

(3) $$\begin{array}{l} S_{C_{1},gas}=\frac{Y_{C_{1},\;gas}}{X_{butanol}} \end{array}$$

(4) $$\begin{array}{l} S_{i}=\frac{F_{i}}{F_{butanol_{in}}X_{butanol}} \end{array}$$

Please note that the maximum selectivity depends on the component, i.e., 4 for C_1_ compounds, 2 for C_2_ compounds, 1 for C_4_ compounds as well as C_3_ compounds, considering that a butanol molecule can deliver max 1 C_3_ molecule, and 12 for H_2_.

## Results

### Catalyst Characterization

#### XRF, Physisorption and Chemisorption

Table 1 shows the results of N_2_ physisorption of both fresh and spent 250–420 μm catalyst. Surface area calculated with the seven-point BET method and the NLDFT model are in agreement, showing a small increase during the experiment. The total pore volume is also increased during the experiment and the pore size distribution shifts to slightly larger pores. The catalyst is mesoporous. Surprisingly, application in APR causes a minor but significant increase in surface area and pore volume, despite the fact that ZrO_2_ is reported to be stable under APR conditions (Elliott et al., 1993). Also, no significant changes in the morphology of ZrO_2_ were observed during preliminary aging experiments in pure water at 200°C for 12 h (not shown).

**Table 1:** N_2_ physisorption results for the 250–420 μm catalyst.

**Sample**	**Surface area (m**^**2**^ **g**^**−1**^**)**				**Pore volume (cm**^**3**^ **g**^**−1**^**)**	
	**7-point BET**		**NLDFT**			
	**Fresh**	**Spent**	**Fresh**	**Spent**	**Fresh**	**Spent**
Rh/ZrO_2_ 250–420 μm	37.0	42.5	36.6	42.4	0.100	0.121

Table 2 shows the Rh loading in the fresh and spent catalysts, as measured by XRF. The Rh leaching increased significantly with increasing dimension of the catalyst particles. The reduction in Rh content is caused by leaching. Please note that any apparent decrease of Rh loading due to catalyst mass being increased by deposit formation is in all cases much smaller than the observed effects, therefore leaching is the main cause of the decreasing Rh loading.

**Table 2:** XRF analysis results.

**Sample**	**Time on stream (min)**	**Rh loading (wt.%)**				
		**Target**	**Fresh**	**Spent**	**Rh loss (%)**	
Rh/ZrO_2_ 40–60 μm	150		0.5	0.43	0.43	0.9
Rh/ZrO_2_ 60–100 μm	150		0.5	0.42	0.40	5.4
Rh/ZrO_2_ 250–420 μm	210		0.5	0.50	0.36	27.7

**Figure 2:**
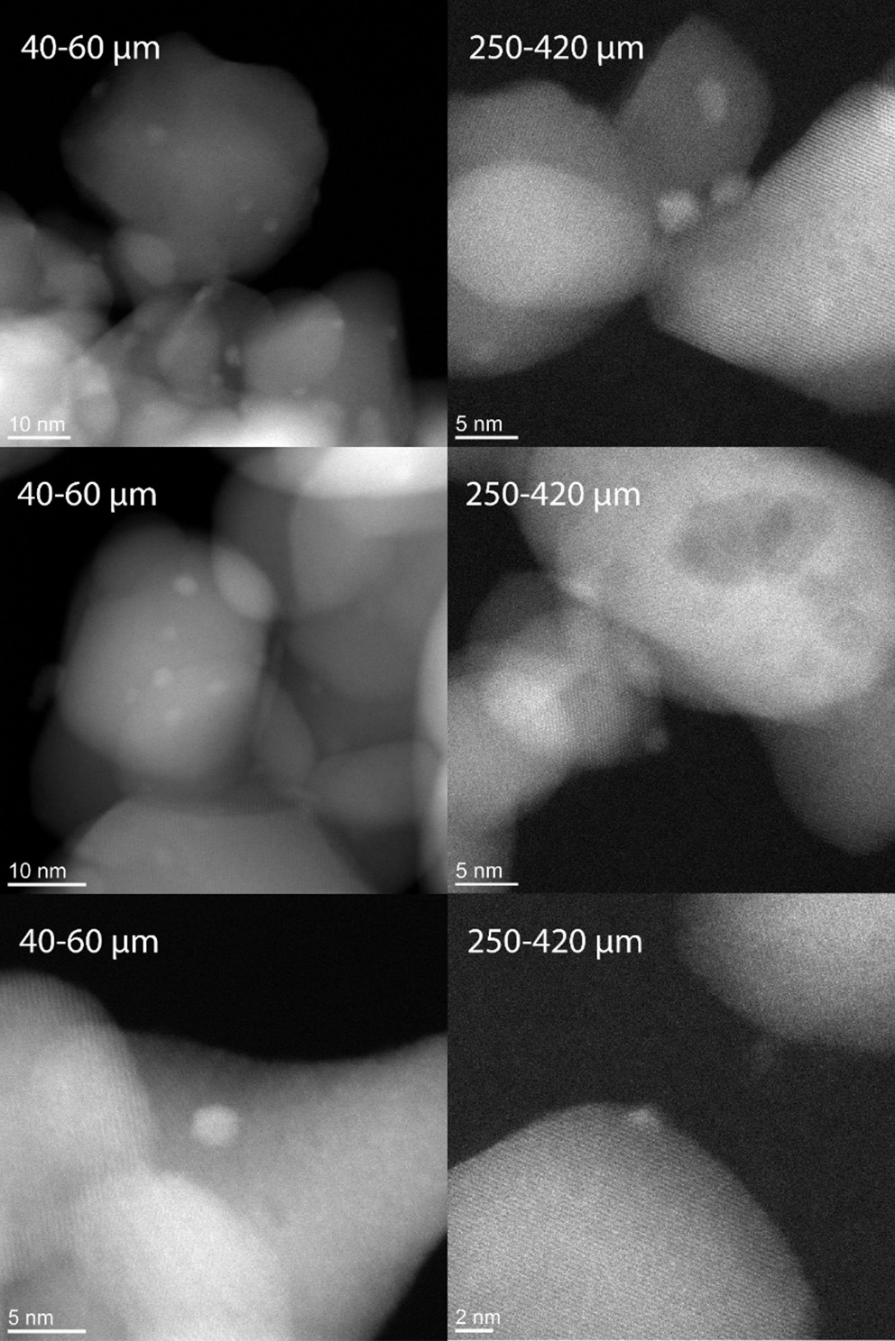
Dark field STEM images of the spent 40–60 and 250–420 μm Rh/ZrO_2_ catalysts.

Table 3 shows the Rh dispersions and metal surface areas in fresh and spent catalysts as measured by H_2_ chemisorption as well as the average Rh crystallite size calculated from both Rh surface area and STEM images, shown in Figure 2. The fresh catalysts show very good dispersion and small Rh crystallite size. During the reaction, the available Rh surface area is reduced by factor of typically 2, without any significant effect of the zirconia particle. The increase in metal particle sizes according chemisorption are in reasonable agreement with the observed particle sizes observed with STEM, presented below.

**Table 3:** H_2_ chemisorption and particle size determined with STEM; the typical experimental relative error in dispersion and surface area is 10%.

**Sample**	**Rh dispersion**		**Rh surface area**		**Average Rh particle size (nm)**		
	**H**_****2****_ **chemisorption (%)**		**H**_****2****_ **chemisorption (m**^****2****^ ${\text{g}}_{\text{catalyst}}^{-1}$**)**		**H**_****2****_ **chemisorption**		**STEM**
	**Fresh**	**Spent**	**Fresh**	**Spent**	**Fresh**	**Spent**	**Spent**
Rh/ZrO_2_ 40–60 μm	78	34	1.5	0.6	1.4	3.3	2.7
Rh/ZrO_2_ 60–100 μm	110	70	2.1	1.2	1.0	1.6	NA
Rh/ZrO_2_ 250–420 μm	64	47	1.4	0.75	1.8	2.4	2.6

#### STEM Analysis of Spent Catalyst

**Figure 3:**
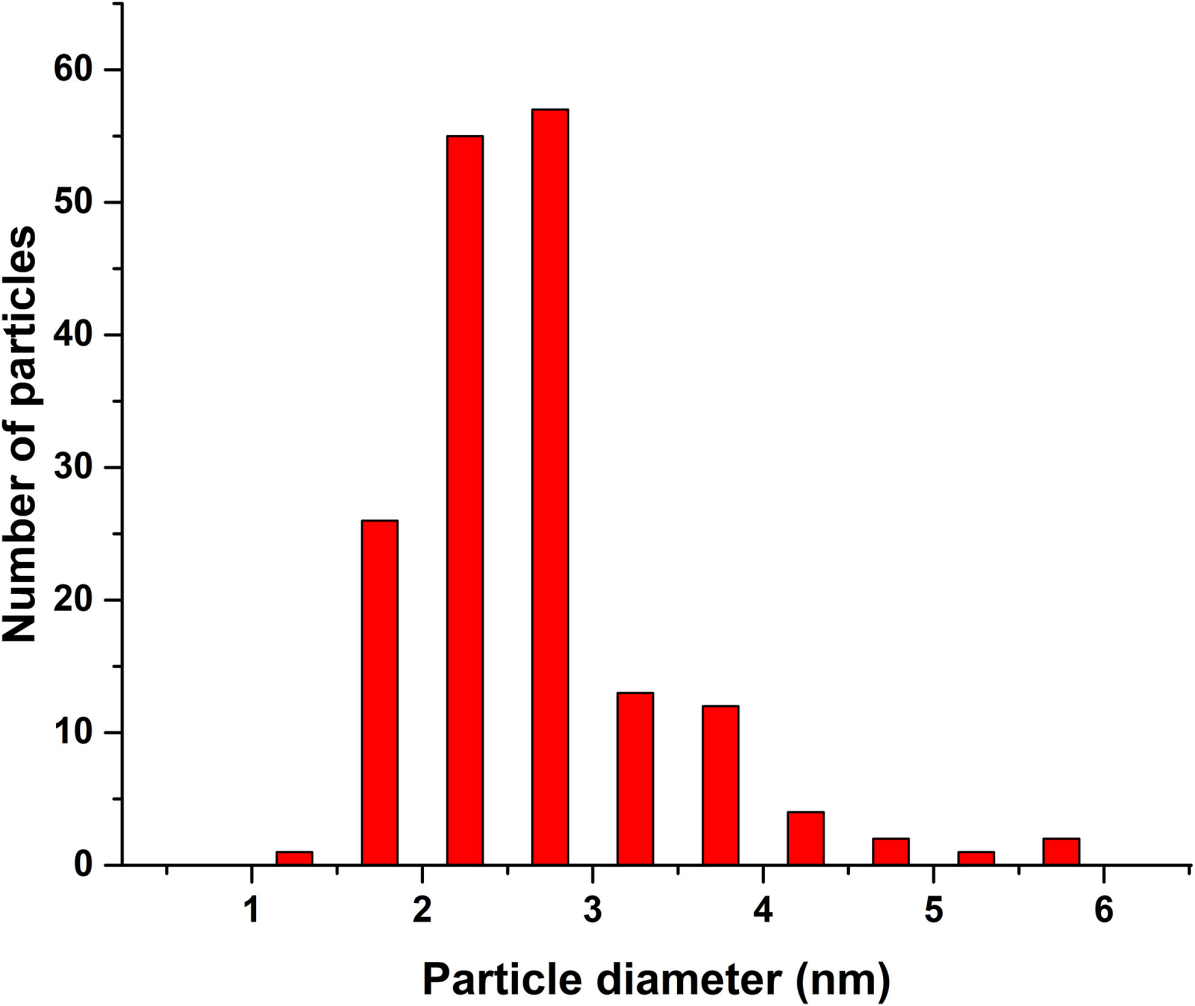
Rh particle size distribution on the 40–60 μm spent catalyst according STEM.

Figure 2 shows dark field STEM images of the spent 40–60 and 250–420 μm catalysts. The Rh crystallites in the fresh catalysts were oxidized, making it impossible to see them with STEM without a reductive pretreatment. Rh in the spent catalysts is found in mostly round and sometimes also oval particles. The particle size distribution is quite narrow, as seen in Figure 3 for the 40–60 μm catalyst. Rh particles were considerably harder to locate in the larger catalyst particles, leading to a somewhat limited data set (30 particles as compared to 173 on the 40–60 μm catalyst). However, the distribution in 250–420 μm catalyst (Supplementary Figure A-1) reveals no significant difference with the distribution shown in Figure 3.

#### Elemental Analysis

**Figure 4:**
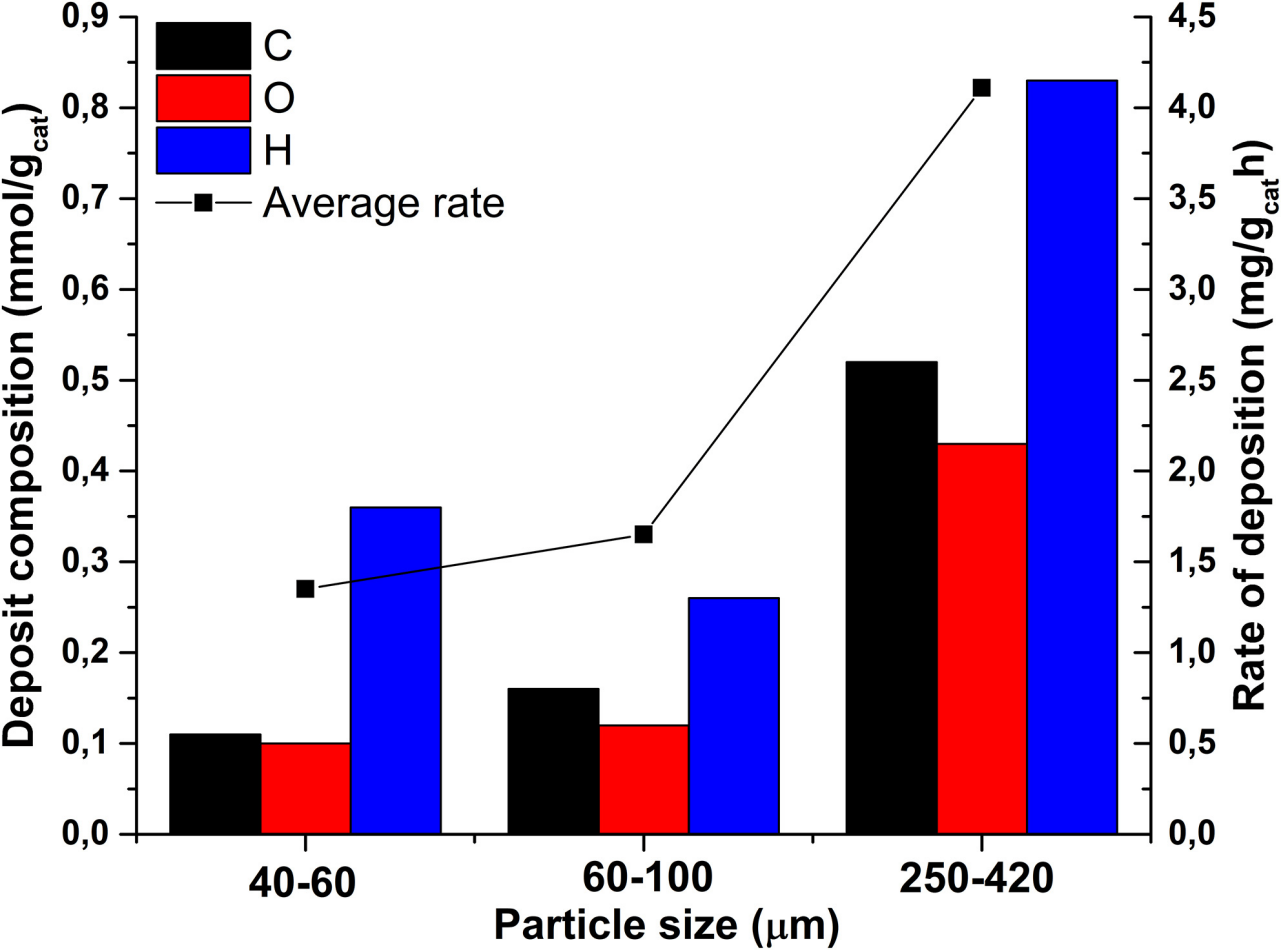
Elemental composition and amounts of deposits as well as averaged rate of formation of deposits in spent catalysts; contamination on fresh catalysts was subtracted.

Figure 4 shows the C, H, and O elemental analysis results of spent catalysts; the amount of deposits was calculated by subtracting the amount of C, H, and O detected on fresh catalyst from the amounts detected on spent catalyst. The average rate of deposition (in mg ${\text{g}}_{{\text{catalysts}}^{-1}}$ h^−1^) is calculated by dividing the amount of deposits by the TOS in order to take into account small differences in the TOS, as also presented in Figure 4. Formation of deposits clearly increases with increasing particle size, particularly the amounts of carbon and oxygen as well as the total mass of deposit (mg ${\text{g}}_{{\text{catalyst}}^{-1}}$) and the average rate of deposition.

### Catalytic Performance

#### Conversion and Products Yields

**Figure 5:**
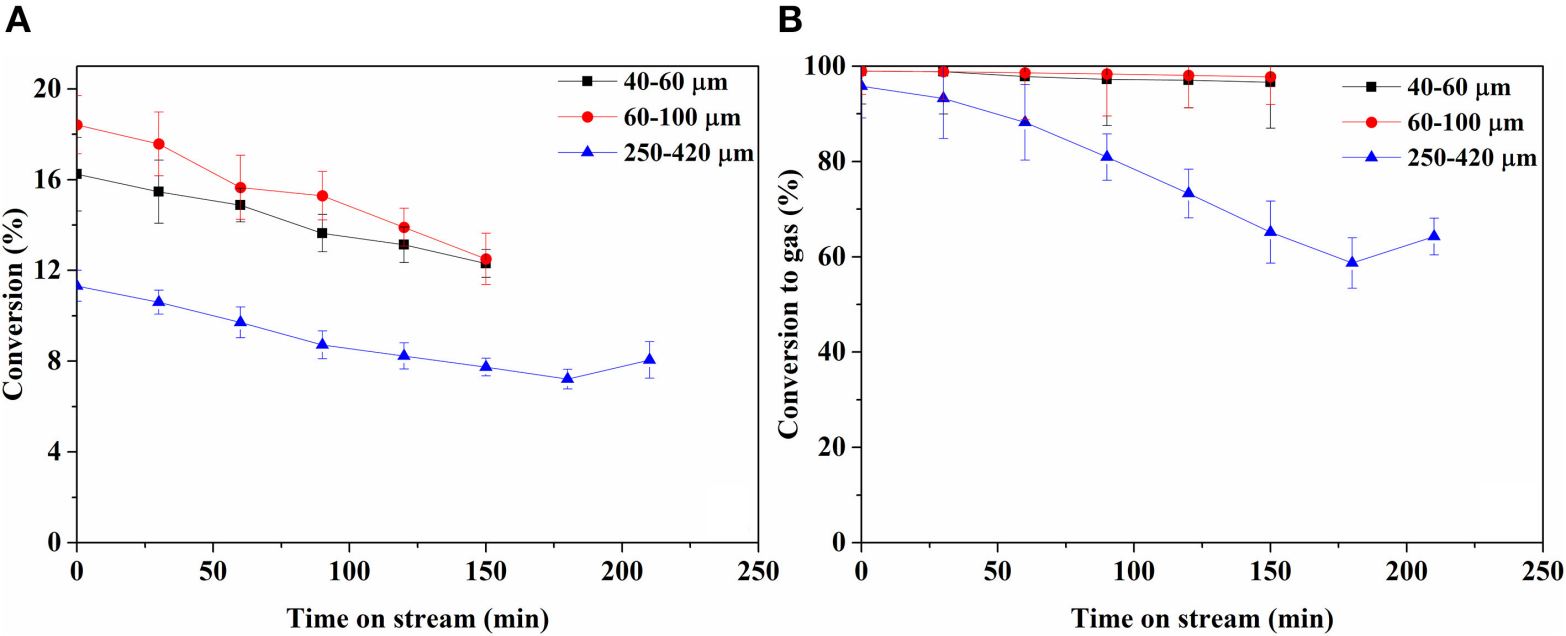
Butanol conversion **(A)** and conversion to gas phase products on C_1_-basis over time **(B)**.

Figure 5 shows conversion and carbon-based selectivity to gaseous product over time. The conversion over the 40–60 and 60–100 μm catalysts is similar, whereas the conversion over the 250–420 μm catalyst is consistently 30–40% lower. Conversion on all catalysts declined at a similar rate over time. Selectivity to gaseous products remained high on 40–60 and 60–100 μm catalysts, declining slowly over time from ca. 99 to 97% in 150 min. On 250–420 μm catalyst, gas selectivity starts similarly high, declining severely over time. The conversion on bare support was too low to enable reliable quantification (Supplementary Figure A-3A).

#### Product Distribution

**Figure 6:**
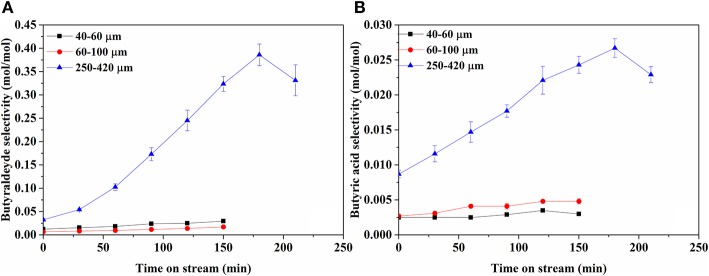
Selectivity of products in liquid phase; **(A)** Butyraldehyde and **(B)** Butyric acid. Note the difference scale between **(A)** and **(B)**.

Figure 6 shows selectivities to butyraldehyde and butyric acid, the main products in liquid phase, over time. In addition, traces of acetic acid and ethanol were detected. On bare support (Supplementary Figure A-3B), only butyraldehyde is observed in very low amounts, decreasing with TOS. Clearly, butyraldehyde selectivity is much higher compared to the butyric acid selectivity. The liquid product selectivities over 40–60 and 60–100 μm catalysts are very similar, increasing slowly with time on stream. In contrast, the 250–420 μm catalyst produces much more butyraldehyde and butyric acid, both increasing significantly with time on stream. The molar ratio of aldehyde to acid also increases over time, particularly on the 250–420 μm catalyst, from an initial value 4 to about 14 by the end of the experiment.

**Figure 7:**
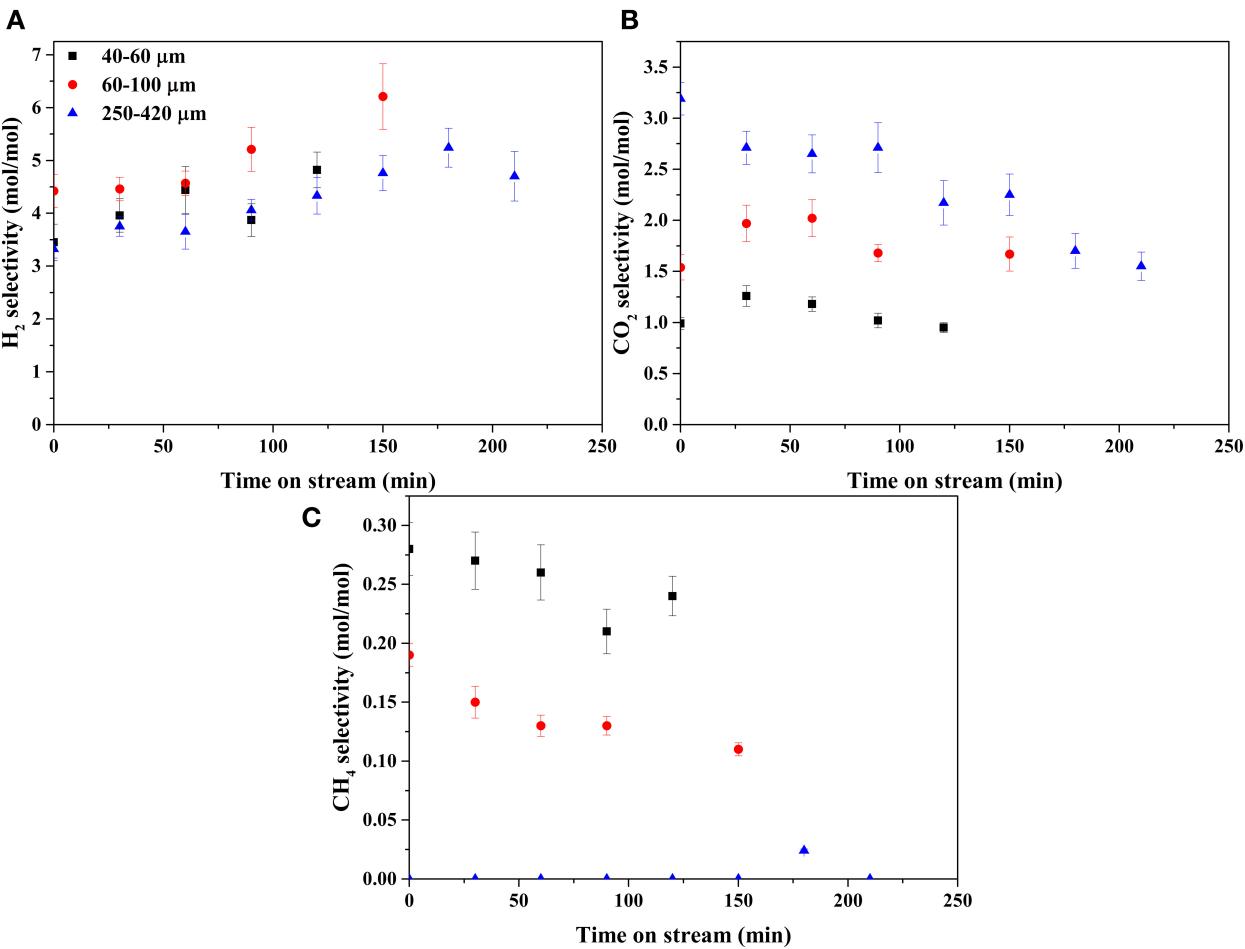
Selectivities of gaseous products: **(A)** H_2_, **(B)** CO_2_, and **(C)** CH_4_.

**Figure 8:**
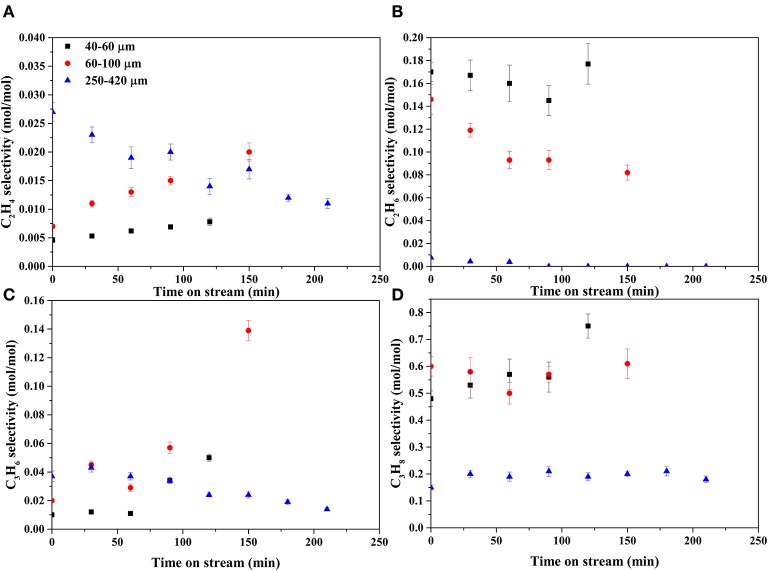
Selectivities of C_2−3_ products. **(A)** ethylene, **(B)** ethane, **(C)** propylene, and **(D)** propane.

Figures 7, 8 show the selectivities to products in the gas phase, i.e., H_2_, CO_2_, and CH_4_ (Figure 7) and C_2−3_ hydrocarbons (Figure 8). The selectivity to CO was always very low, below 0.05 mol mol^−1^ (Supplementary Figure A-2). On the bare support formation of H_2_, CO_2_, and C_3_ was detected at such low concentration that quantification was not possible. H_2_ selectivities (Figure 7A) were similar on all catalysts, showing no effect of the catalyst particle size. CO_2_ selectivities (Figure 7B) decreased with decreasing size in the order 250–420 > 60–100 > 40–60. The CO_2_ selectivity decreased over time on the 250–420 μm catalyst, whereas such a trend is less clear on the smaller catalysts. Formation of methane (Figure 7C) increased with decreasing particle size, opposite to CO_2_. In fact, the 250–420 μm catalyst produced hardly any methane. Also selectivity to C_2_ compounds (Figures 8A,B) increased with decreasing catalyst particle size, similar to methane. Furthermore, ethane (Figure 8B) was the main C_2_ product over the 40–60 and 60–100 μm catalysts, while the 250–420 μm catalyst produced more ethylene than ethane (Figure 8A). Propane (Figure 8D) was the main C_3_ product on all catalysts, despite the relatively large scatter in the data. Furthermore, large catalyst particles (250–420 μm) produce less C_3_ compounds than the 40–60 and 60–100 μm catalysts, showing similar C_3_ selectivities.

## Discussion

### Mass Transfer Criteria

The significance of external mass transfer was evaluated using the Mears' criterion (Equation 5; Mears, 1971):

(5) $$\begin{array}{l} C_{M}=\frac{r_{A}^{\prime }\rho_{b}Rn}{k_{c}C_{Ab}}<0.15 \end{array}$$

The detailed calculation is presented in the Supplementary Material. The highest value obtained for the most active and largest catalyst particles was 2.3^*^10^−3^, leading to the conclusion that external mass transfer limitations can be disregarded.

The significance of internal mass transfer was estimated using the Weisz-Prater criterion (Equation 6; Weisz and Prater, 1954). Assuming first order reaction, the criterion becomes:

(6) $$\begin{array}{l} C_{WP}=\frac{r_{A}^{\prime }R^{2}\rho_{c}}{D_{e}C_{As}}<0.25 \end{array}$$

**Figure 9:**
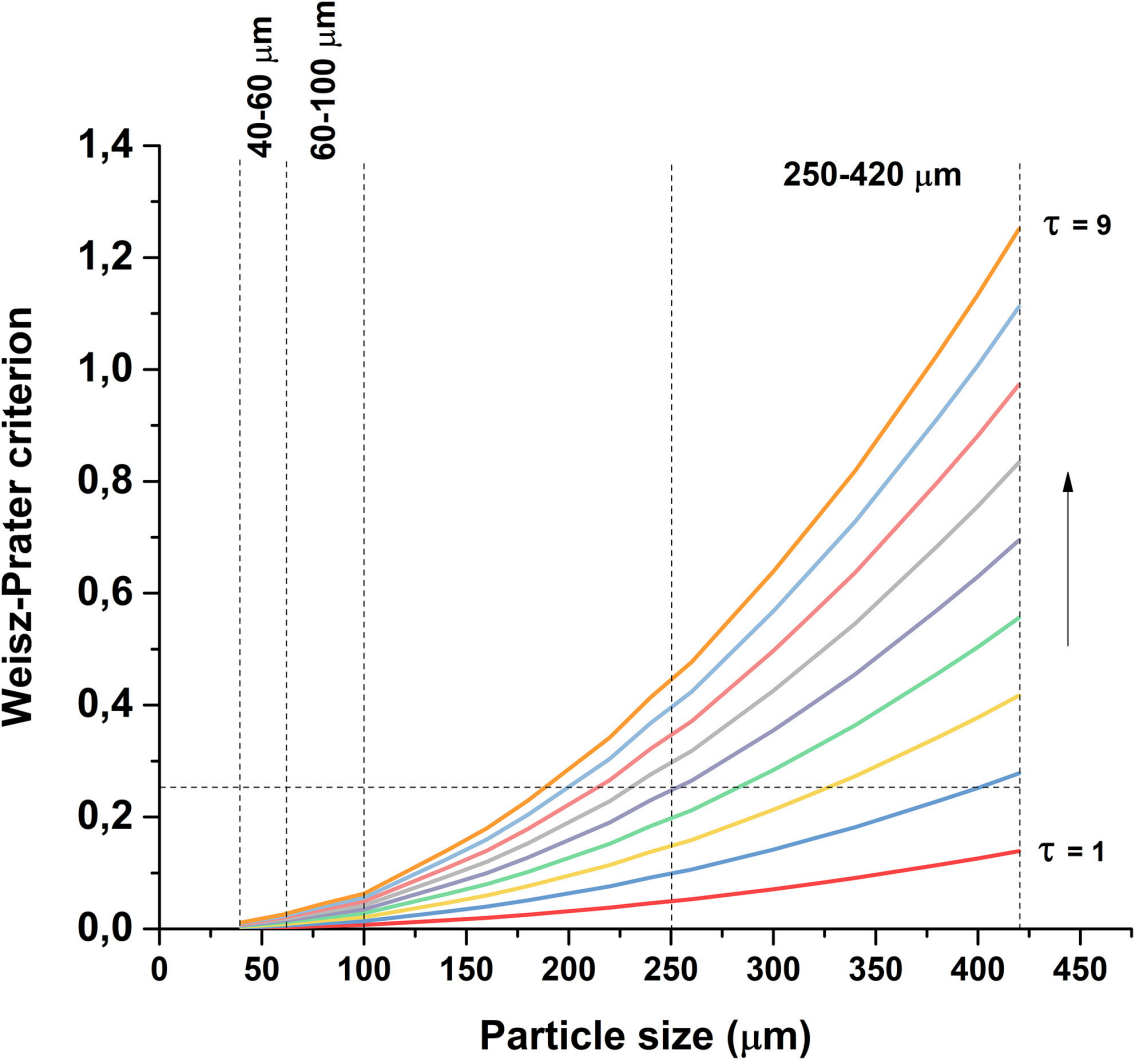
Value of Weisz-Prater criterion as a function of particle size and tortuosity factor.

In which *r'*_*A*_ is the observed reaction rate (kmol_butanol_ ${\text{kg}}_{{\text{cat}}^{-1}}$ s^−1^), *R* is the catalyst particle radius (m), ρ_*c*_ is the density of the catalyst (kg m^−3^), *C*_*As*_ is the concentration of butanol on the external catalyst surface (kmol m^−3^) and *D*_*e*_ is the effective diffusion coefficient (m^2^ s^−1^), calculated according to Equation (7):

(7) $$\begin{array}{l} D_{e}=\frac{D_{AB}\phi_{p}}{\tau } \end{array}$$

in which *D*_*AB*_ is the binary diffusion coefficient of butanol in water (m^2^ s^−1^), ϕ_*p*_ is the catalyst pellet porosity and τ is the tortuosity factor. Catalyst density ρ_*c*_ and porosity ϕ_*p*_ were estimated using the bulk density of non-porous ZrO_2_ and the measured pore volume of the catalyst. As the external mass transfer was considered not limiting, the concentration at the external catalyst surface was assumed to be equal to the bulk concentration, *C*_*As*_ ≈ *C*_*Ab*_. Since the exact value of the tortuosity factor was unknown, the value of C_WP_ was calculated as a function of catalyst particle size with values of τ ranging from 1 to 9, shown in Figure 9, using the highest observed reaction rate at time zero. As clearly inferred from the graph, internal diffusion is not limiting for the two smaller particle sizes, but may be limiting for the largest particle size, depending on the value of the tortuosity factor. Therefore, the role of internal diffusion required further experimental evaluation, as will be discussed later. In order to discuss the effect of internal transport on the product distribution, it is necessary to discuss first the main reactions contributing to the overall conversion.

### Catalyst Performance; Comparison to Literature

APR of alcohols and polyalcohols has been widely investigated, in particular with C-to-O ratio of 1:1 (Shabaker et al., 2003a; Kirilin et al., 2010, 2014). Ethanol, propanol or butanol were typically less studied; the presence of the alkyl groups makes complete reforming less facile and formation of alkanes is reported.

Lobo et al. (2012) studied APR of *n*-propanol over Pt catalysts (5 wt.% propanol solution, 250°C temperature, 69 bar pressure), reporting reaction rates per gram catalyst about one order of magnitude higher. Unfortunately, the Pt loading is not reported. Ethane and carbon dioxide are the main products, with a ratio close to 1, whereas propanal found in the liquid phase.

Godina et al. studied the reforming of alcohols with 3 carbon atoms using Pt-based catalyst on polymer based spherical activated carbons (Godina et al., 2018). Even in this case, exclusively propanal and propionic acid are reported in the liquid phase, whereas ethane was the most formed alkane. On the other hand, methane was not detected, in contrast to our results in the case of the smaller catalyst particles. The presence of propanal and propionic acid from 1-propanol APR was also confirmed by Wawretz and co-workers (Wawrzetz et al., 2010) with an alumina supported Pt catalyst.

Coronado et al. studied the APR of ethanol and propanol with nickel based catalysts on ceria-zirconia supports (Coronado et al., 2018, and literature cited therein), reporting propanal as main liquid product, together with a small amount of propionic acid. On the other hand, methane was found in the gas phase, which was attributed to methanation of CO and CO_2_.

Pipitone et al. (2019) studied the aqueous phase reforming of butanol with a Pt-based catalyst in a batch system. The produced gas phase contained hydrogen carbon-dioxide and propane in a 2-1-1 molar ratio, in accordance with the previous works on C3 alcohols (Godina et al., 2018). On the other hand, the nature of the active sites seems to have an effect on the product distribution in the gas phase. Indeed, Roy et al. (2011, 2014) studied APR of *n*-butanol over alumina and ceria supported Ni catalysts (20 wt.% Ni loading, 5 wt.% butanol solution, 185–215°C and 10–31 bar), resulting in reaction rates an order of magnitude lower. The reported product distribution on Ni is similar to the present work, with the exception of somewhat higher selectivity to C_2_ hydrocarbons, which the authors attributed to Fischer-Tropsch reactions. The main reaction pathway was proposed to proceed through consecutive dehydrogenation and decarbonylation steps, very similar to the findings of Lobo et al. for APR of propanol with Pt catalysts (Lobo et al., 2012). Tishchenko coupling of propionaldehyde followed by hydrolysis and propionic acid decarboxylation was observed as a side reaction pathway on Pt. Moreover, it was suggested that propane was reformed to hydrogen and carbon monoxide, as likely occurred also in the reaction conditions reported during the present investigation. This result may be explained by the higher activity of nickel toward C-C bond breaking compared to platinum (Sinfelt, 1973).

### Reaction Scheme for Rh Catalyst

**Figure 10:**
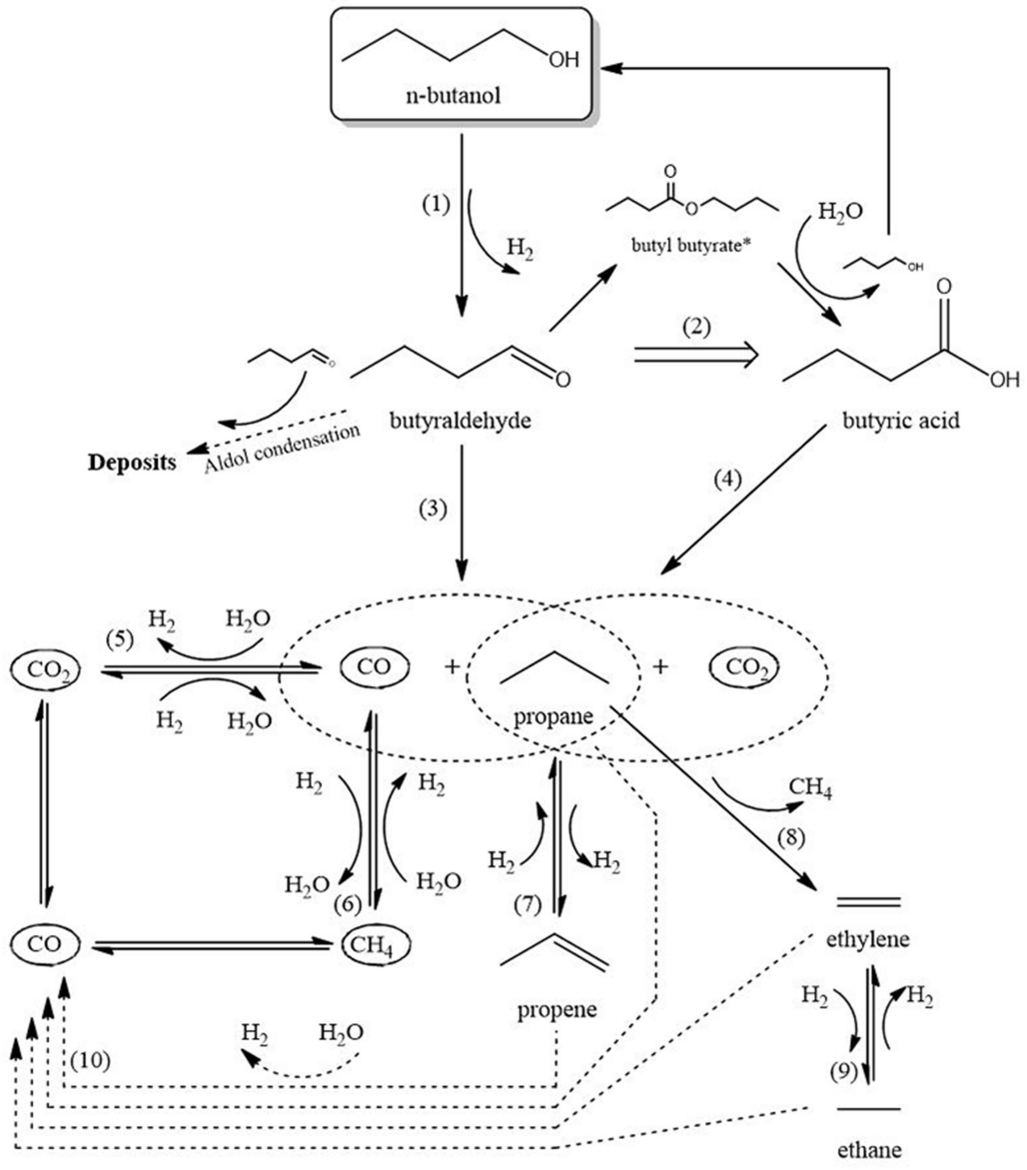
Schematic reaction network. Products marked with *not observed during the experiments.

As no reaction scheme has been reported so far for APR of butanol over Rh catalysts, a proposal for such a scheme is described below (Figure 10), based on the observations described above and inspired by literature on APR of butanol on Ni catalysts (Roy et al., 2011, 2014) and of 1-propanol on Pt catalysts (Lobo et al., 2012). Please note this scheme is required for discussing the effect of mass transfer limitations on the product distribution. The scheme is divided in three stages: (I) initial conversion, (II) initial gas formation, and (III) hydrocarbon reactions and reforming.

Stage I is the initial conversion of butanol to liquid intermediates via reactions 1 and 2. Reaction 1, dehydrogenation of butanol into butyraldehyde, mostly takes place on Rh. Figure 6A shows a significant yield of butyraldehyde over the Rh catalyst, whereas the yield over plain zirconia (Supplementary Figure A-3B) is <0.5 mol% of butanol. This agrees with literature, where dehydrogenation of alcohols is mostly reported on metals (Roy et al., 2011, 2014; Lobo et al., 2012), although it is also reported on zirconia (Sabatier and Mailhe, 1910; Shinohara et al., 1997). Interestingly, the butyraldehyde selectivity (Figure 6A) increases over time and the selectivity to gas products (Figure 5B) declines simultaneously. This is especially clear over the 250–420 μm catalyst, indicating that the reactions forming gasses deactivate more than the dehydrogenation reaction. Furthermore, no C_3_ oxygenates were observed in the liquid phase. Therefore, reactions involving C-C cleavage in butanol are not significant and reaction via butyraldehyde dominates, in agreement with suggestions in literature for Ni and Pt (Roy et al., 2011, 2014; Lobo et al., 2012). Reaction 2 is the formation of butyric acid, most likely via Tishchenko esterification on Rh, supported by the fact that butyric acid selectivity closely follows the selectivity for butyraldehyde (Figures 6A,B). The intermediate butyl-butyrate was not observed, as would be expected considering that in aqueous medium the equilibrium strongly favors hydrolysis of butyl-butyrate.

Stage II is the formation of CO_x_ according reactions 3 and 4, respectively, decarbonylation and decarboxylation. The claim that the C-C bond is cleaved via decarbonylation and/or decarboxylation is supported by the observations that propane and C_4_ oxygenate products are the dominant products, as well as by the absence of any C_3_ oxygenates and the virtual absence of C_2_ oxygenates. Decarbonylation of butyraldehyde (reaction 3) is the main reaction forming gaseous products, as much more butyraldehyde than butyric acid is formed (Figure 6). The reaction mainly takes place on the metal, but can also be observed on the bare support (Supplementary Figure A-3A), in agreement with literature (Bie et al., 2013; Miao et al., 2016). Decarboxylation of butyric acid (reaction 4) requires the presence of an active metal according literature (Bie et al., 2013; Miao et al., 2016).

Stage III consists of the reactions of hydrocarbons (reforming and (de-)hydrogenation) and (R-)WGS (reactions 5–10). The water-gas-shift reaction (WGS, reaction 5) converts virtually all CO to CO_2_ and H_2_. The aqueous environment is expected to strongly drive WGS toward the products (Shabaker et al., 2003b), causing extremely low CO yields (Supplementary Figure A-2).

Propane produced in reactions 3 and 4 can be dehydrogenated over Rh (reaction 7) as indicated by the formation of propylene in Figure 8C. The shorter C_1−2_ hydrocarbons are likely formed via propane hydrogenolysis (reaction 8). Although propane hydrogenolysis has not been reported in aqueous phase, it is known to be readily catalyzed by Rh in this temperature range in gas phase (Bond et al., 1996).

Methanation (reaction 6) has been reported on Rh in APR (Davda et al., 2003); however, methanation is slow at the low temperature of operation (Mutz et al., 2015). We speculate that the dominant pathway to methane is hydrogenolysis of propane based on the observation that the methane selectivity (Figure 7C) is very similar to the C_2_ selectivity (sum of ethane and ethylene in Figures 8A,B), consistent with formation of methane, ethane and ethylene according reactions 8 and 9. Interestingly, selectivity of both methane and C_2_ increase when operating with smaller catalyst particles.

Reforming of hydrocarbons (an aggregate reaction 10) probably contributes, possibly via the activation mechanism discussed above, based on three observations. First, on all catalysts the selectivity to CO_2_ (Figure 7B) is higher than 1 mol/mol butanol, indicating that next to reactions 3 and 4 an additional pathway to CO_2_ must exist. Second, H_2_ formation via exclusively the sequence dehydrogenation-decarbonylation-WGS (reactions 1, 3, 5) would result in selectivity to hydrogen of 2 mol per mole butanol converted. Figure 7A shows that the H_2_ selectivity varies between 3 and 6, providing clear evidence that an additional pathway contributes significantly. Third, the combined selectivity of C_2−3_ products (Figure 8) is <1 mol per mol butanol converted, clearly indicating that C_2−3_ products are being consumed in a consecutive reaction, most likely reforming. In addition to experimental observations, Rh is more active for steam reforming at low temperature compared to e.g., Pt or Ni (Kolb et al., 2004; Halabi et al., 2010), supporting the suggestion that reforming of alkanes contributes at the temperatures used in the present work. Further research would be needed to confirm this hypothesis.

### Effect of Mass Transfer

Internal mass transfer can affect the local concentration of reactants as well as intermediate reaction products, possibly affecting the consecutive reactions and therefore the final product distribution. The data shows several clear trends as discussed below.

Butanol conversion is limited by diffusion in large catalyst particles. Figure 5A shows that conversion of butanol over the 250–420 μm catalyst is lower than over the smaller catalyst particles, whereas the conversion is similar over both of the smaller particle sizes. This indicates that diffusion limits the reaction on large catalyst particles, in agreement with the estimated values of Weisz-Prater criterion in Figure 9. Possibly, subtle differences in the metal dispersion, metal loading and metal surface area (Table 3) cause the relative high activity of the 60–100 μm fraction. On the other hand, difference in performance between the 40–60 μm fraction and the 250–420 μm fraction are clearly due to mass transfer effects.

Butyraldehyde coupling is enhanced on large particles as the selectivity to butyric-acid is higher on the larger particles (Figure 6B). The Tishchenko coupling reaction (reaction 2) is 2nd order (Anderson and Peters, 1960) and thus rates are strongly influenced by the butyraldehyde concentration. Slow diffusion increases the local concentration of butyraldehyde in the center of the catalyst particles, thus resulting in high butyric-acid yield.

Conversion of hydrocarbons is enhanced on large catalyst particles, including dehydrogenation (reaction 7), hydrogenolysis (reaction 8), and reforming (reactions 6, 10). This is based on three observations. First, the selectivity to methane (Figure 7C) and C_2−3_ hydrocarbons (Figure 8) is the lowest on large catalyst particles, suggesting more reforming, attributed to sluggish diffusion of dissolved C_1, 2, 3_ hydrocarbons. Second, the ratio of olefins to alkanes is at the same time higher on the large catalyst particles, indicative for more dehydrogenation. Third, CO_2_ selectivity (Figure 7B) is the highest over the large catalyst particles. Furthermore, the CO_2_ selectivity is well above 1 mol per mol butanol converted, especially on large catalyst particles (Figure 7B), also indicating reforming activity as discussed above. The products of reactions 3 and 4 (i.e., CO, CO_2_, and propane) are all gasses and diffusion out of the catalyst particles is controlled by diffusion of these molecules dissolved in water. However, the H_2_ selectivity decreases with catalyst particle size (Figure 7A) despite reforming activity of large particles, indicating that longer diffusion length increases consecutive reactions consuming hydrogen more than consecutive reactions producing hydrogen.

This argument is analogous to the mechanism proposed by Neira D'Angelo et al. (2013), accounting for decreasing H_2_ yield in case of mass transfer limitation because of the lower selectivity, while the conversion is not strongly affected. The difference in the operation of the reactors should be noted. In our case, we do not co-feed any gas, and formation of gas phase can occur only via nucleation of oversaturated solutions. In the case of Neira D'Angelo et al. (2013) as well as most APR studies in continuous operation, inert gas is added to the reactant stream and the reactor operated in trickle-phase mode, resulting in stripping of gaseous components in the reactor. This will enhance internal diffusion by decreasing the concentration at the external surface of the catalyst particles, i.e., in the bulk of the liquid. Therefore, our experiments are more sensitive for effects of internal diffusion of intermediate products.

The particle size affects not only the liquid and gaseous products distribution, but also strongly influences the stability of the catalyst. As reported in Figure 5B, the gas selectivity declined slowly for the 40–60 and 60–100 μm catalysts, while decreasing significantly for the 250–420 μm catalyst. This can be understood considering the high butyraldehyde concentration present inside the large catalyst particles. The aldehyde is a precursor of deposits via aldol-condensation reactions enhanced by the acid-base properties of the zirconia support, causing deactivation of the catalyst (Takanabe et al., 2006; Koichumanova et al., 2018). This hypothesis is also supported by the fact that larger particles form more deposits (Figure 4) and the high oxygen and hydrogen content of the deposits agrees with formation via butyraldehyde condensation, similar to results with the same catalyst system in gas phase reforming (Harju et al., 2016). Furthermore, it is also clear that larger support particles suffer more from Rh leaching than small particles (Table 2) which is in line with the fact that the formation of butyric acid is enhanced, resulting in more acidic conditions inside the larger catalyst particles, enhancing leaching.

Finally, it should be noted that description of internal mass transfer according Thiele modulus and Weisz-Prater criterion can be criticized for the case of APR. These models assume molecular diffusion of dissolved species in pores filled with water. However, APR produces molecules (H_2_, CO, CO_2_, CH_4_, and other light hydrocarbons) that are forming a new phase during the reaction, i.e., gas phase. The critical question is whether bubbles form inside or outside the catalyst particles and at this time this question remains unanswered. Theoretical work of Datsevich (2003a; 2003b; 2004; 2005, Oehmichen et al., 2010) has shown that formation of gas bubbles can influence transfer inside particles dramatically, whereas we have recently demonstrated in a microfluidic device, mimicking pores in a catalyst support, that both retardation as well as enhancement of transport on the pore is possible (Espinosa et al., 2018). Very recently, a simulation using APR of glycerol as model reaction showed the effect of bubbles on the kinetics and transport phenomena in a 2D system (Ripken et al., 2019). This clearly needs further research to decide if such effects experimentally contribute in practical 3D porous catalysts.

## Conclusions

The influence of internal mass transfer in Rh/ZrO_2_ catalysts for APR of 1-butanol was studied by varying the support particle size. Larger support particles cause a minor but significant decrease in activity, which can be attributed to internal mass transfer limitations. The effect on selectivity and stability is much stronger though.

A reaction scheme is proposed in order to discuss the effects of internal mass transfer on product distribution and stability. The reaction scheme includes three main stages i.e., (1) formation of liquid intermediates via dehydrogenation, (2) formation of gas via decarbonylation/decarboxylation reactions and (3) hydrocarbon hydrogenolysis/reforming/dehydrogenation. The main pathway to hydrogen involves stages 1 and 2, while methane, ethane, and ethylene are formed via hydrogenolysis of propane.

Internal mass transport limitation of butyraldehyde enhances formation of butyric acid. Slow diffusion of methane, ethane, and propane dissolved in water promotes consecutive reactions, i.e., dehydrogenation, hydrogenolysis, and reforming. Furthermore, large support particles deactivate faster, attributed to high concentrations of butyraldehyde inside the catalyst particles, enhancing deposit formation via aldol condensation reactions, as well as to local high acidity caused by butyric acid.

## Data Availability Statement

The datasets generated for this study are available on request to the corresponding author.

## Author Contributions

HH and LL: conceptualization, methodology, validation, formal analysis, investigation, project administ, and ration. LL: resources, supervision, and funding acquisition. HH, GP, and LL: data curation and writing—review and editing. HH: writing—original draft preparation.

### Conflict of Interest

The authors declare that the research was conducted in the absence of any commercial or financial relationships that could be construed as a potential conflict of interest.
