# Supplementary material for: Influence of the Catalyst Particle Size on the Aqueous Phase Reforming of n-Butanol Over Rh/ZrO2
Source: Front Chem. 2020 Jan 28;8:17. doi: 10.3389/fchem.2020.00017 (PMC6997294; doi:10.3389/fchem.2020.00017)
Supplement: Supplementary file 1 [file Table_1.DOCX]

Supplementary Material

# Supplementary Data

The significance of external mass transfer was evaluated using the Mears’ criterion (Mears, 1971):

$C_{M}=\frac{r_{A}^{'}\rho_{b}Rn}{k_{c}C_{Ab}}<0,15$ (6)

In which *r’_A_* is the measured reaction rate (kmol kg_cat_^-1^ s^-1^), *ρ_b_* is the bulk density of catalyst bed (kg m^-3^), *R* is the particle radius (m), *n* is the reaction order, *C_Ab_* is the bulk butanol concentration (kmol m^-3^) and *k_c_* is the mass transfer coefficient (m s^-1^) derived from the empirical correlation for mass transfer in fixed and fluidized beds by Dwlvedi and Upadhyay (Dwlvedi and Upadhyay, 1977), which is valid for both gases (Re>10) and liquids (Re>0.01):

$\phi J_{D}=\frac{0.765}{Re^{0.82}}+\frac{0.365}{Re^{0.386}}$ (7)

In which *ϕ* is the void fraction of the bed, calculated as the ratio of bulk density of the bed and the density of the catalyst, *J_D_* is the Colburn *J* factor and *Re* is the Reynolds number. Generally:

$J_{D}=\frac{Sh}{Sc^{\frac{1}{3}}Re}$ (8)

$Sh=\frac{k_{c}d_{p}}{D_{AB}}$ (9)

$Re=\frac{Ud_{p}\rho}{\mu}$ (10)

$Sc=\frac{\mu}{\rho D_{AB}}$ (11)

Wherein *Sh* is the Sherwood number, *Sc* is the Schmidt number, *d_p_* is particle diameter (m), *D_AB_* is the binary diffusion coefficient of butanol in water (m^2^ s^-1^), *U* is the superficial fluid velocity through the bed (m s^-1^), *ρ* is the density of the fluid and *µ* is the viscosity of the fluid (kg m^-1^ s^-1^). For simplicity, the catalyst particles were assumed spherical in the calculations. As the reaction mixture concentration was low, the viscosity and density of the mixture was approximated as the values for pure water. The viscosity was obtained from a correlation as a function of temperature in DIPPR (DIPPR Project 801 - Full Version) and the density from a correlation as a function of pressure and temperature in the region 1 of the IAPWS-IF97 Formulation (IAPWS-IF97). The diffusion coefficient was estimated using a correlation from Yaws' Transport Properties of Chemicals and Hydrocarbons (Electronic Edition) (Yaws). The reaction was assumed to be first order, which is reasonable considering published data for glycerol(Wawrzetz et al., 2010). Using the highest observed reaction rate at time zero, the criterion value becomes 2.3*10^-3^, 3.9*10^-4^ and 1.7*10^-4^ respectively for 420 µm, 100 µm and 60 µm particles in their respective flow rates. These values are all orders of magnitude lower than the criterion limit 0.15, indicating that external diffusion should have no effect on the conversion rate of butanol. The correlation for the diffusion coefficient was reported to only be valid up to 120 °C temperature, but the values for the criterion are so low that any conceivable changes in the value of the diffusion coefficient don’t cause significant effect.

Figure A‑1. Rh particle size distribution on the 250-420 µm spent catalyst according STEM.

Figure A‑1 shows the particles size distribution in STEM analysis over the spent 250-420 µm catalyst. As noted, the total number of particles counted is low and the size distribution is very similar to 40-60 µm catalyst.

Figure A‑2. Selectivity to CO

Figure A‑2 shows the selectivity (mol/mol) to CO. As noted in the text, CO selectivity is low.


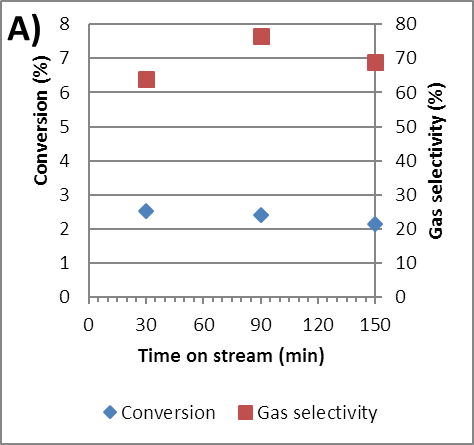

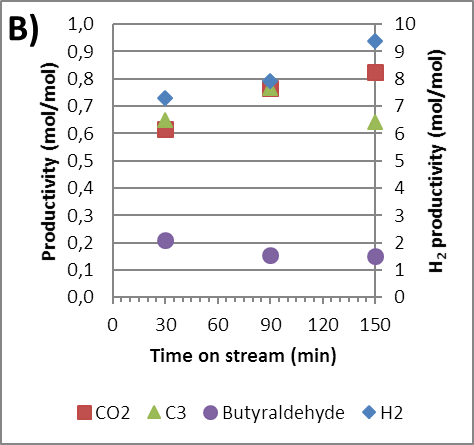


Figure A‑3. A) Butanol conversion and selectivity to gas phase products over the bare support. B) Product selectivities over the bare support

Figure A‑3 shows the butanol conversion as well as selectivity to gas and relevant products. As noted in the text, the very low conversion made reliable quantification of products difficult and the results can be considered indicative at best.

**References**

DIPPR Project 801 - Full Version.

Dwlvedi, P. N., and Upadhyay, S. N. (1977). Particle-Fluid Mass Transfer in Fixed and Fluidized Beds. *Ind. Eng. Chem. Process Des. Dev.* 16, 157–165. doi:10.1021/i260062a001.

IAPWS-IF97 *Int. Assoc. Prop. Water Steam*.

Mears, D. E. (1971). Tests for Transport Limitations in Experimental Catalytic Reactors. *Ind. Eng. Chem. Process Des. Dev.* 10, 541–547. doi:10.1021/i260040a020.

Wawrzetz, A., Peng, B., Hrabar, A., Jentys, A., Lemonidou, A. A., and Lercher, J. A. (2010). Towards understanding the bifunctional hydrodeoxygenation and aqueous phase reforming of glycerol. *J. Catal.* 269, 411–420. doi:10.1016/j.jcat.2009.11.027.

Yaws, C. L. Yaws’ Transport Properties of Chemicals and Hydrocarbons (Electronic Edition). *Knovel*.
